# Walking through Apertures in Individuals with Stroke

**DOI:** 10.1371/journal.pone.0170119

**Published:** 2017-01-19

**Authors:** Daisuke Muroi, Yasuhiro Hiroi, Teruaki Koshiba, Yohei Suzuki, Masahiro Kawaki, Takahiro Higuchi

**Affiliations:** 1 Department of Health Promotion Science, Tokyo Metropolitan University, Tokyo, Japan; 2 Department of Rehabilitation, Kameda Medical Center, Chiba, Japan; Fondazione Santa Lucia Istituto di Ricovero e Cura a Carattere Scientifico, ITALY

## Abstract

**Objective:**

Walking through a narrow aperture requires unique postural configurations, i.e., body rotation in the yaw dimension. Stroke individuals may have difficulty performing the body rotations due to motor paralysis on one side of their body. The present study was therefore designed to investigate how successfully such individuals walk through apertures and how they perform body rotation behavior.

**Method:**

Stroke fallers (n = 10), stroke non-fallers (n = 13), and healthy controls (n = 23) participated. In the main task, participants walked for 4 m and passed through apertures of various widths (0.9–1.3 times the participant’s shoulder width). Accidental contact with the frame of an aperture and kinematic characteristics at the moment of aperture crossing were measured. Participants also performed a perceptual judgment task to measure the accuracy of their perceived aperture passability.

**Results and Discussion:**

Stroke fallers made frequent contacts on their paretic side; however, the contacts were not frequent when they penetrated apertures from their paretic side. Stroke fallers and non-fallers rotated their body with multiple steps, rather than a single step, to deal with their motor paralysis. Although the minimum passable width was greater for stroke fallers, the body rotation angle was comparable among groups. This suggests that frequent contact in stroke fallers was due to insufficient body rotation. The fact that there was no significant group difference in the perceived aperture passability suggested that contact occurred mainly due to locomotor factors rather than perceptual factors. Two possible explanations (availability of vision and/or attention) were provided as to why accidental contact on the paretic side did not occur frequently when stroke fallers penetrated the apertures from their paretic side.

## Introduction

Stroke is a disease caused by infarction, or hemorrhages of the blood vessels in the brain. Stroke is the major cause of neurological disabilities that affect many aspects of daily living. As one such issue, individuals with stroke often exhibit impaired walking, primarily due to motor paralysis on one side (typically the contralateral side of the affected side of the brain) of their body. A typical symptom indicating impaired walking is gait asymmetry. Gait asymmetry is the irregular coordination between the lower limbs and is produced mainly by differences in the magnitude of force displayed between the paretic and non-paretic limbs [1]. Walking with gait asymmetry is biomechanically inefficient for achieving forward progression and makes maintaining balance more challenging [2,3].

A particularly challenging aspect of maintaining balance becomes much more evident during adaptive locomotion, i.e., when basic movement patterns need to be modified adaptively in response to environmental constraints. Previous studies have shown that, as compared to control individuals, stroke individuals had difficulty stepping over an obstacle [4], walking fast while performing a cognitive task concurrently [5], changing their walking speed in response to changes in the optic flow [6], changing direction while walking [7–9], and turning [10,11]. In fact, the risk of falling is likely to increase when stroke individuals turn [12–14].

In line with these studies, the present study was designed to uncover challenging aspect of maintaining during adaptive locomotion in stroke individuals. The uniqueness of this study was to test their ability to safely walk through apertures. Adaptive modification of walking through a narrow aperture includes fine-tuning the walking direction toward the center of the aperture [15,16], decrease in movement speed [17,18], and changes in body configuration such as (upper-) body rotation in the yaw dimension [17–26]. The most powerful means to avoid accidental contact is the body rotation because it effectively reduces horizontal space required for crossing. Testing the ability to safely walk through an aperture has helped not only to understand perceptual-motor control of adaptive locomotion for obstacle avoidance [17,25–27] but also to describe the reason that controlling adaptive locomotion is difficult for some types of participants. Older adults had more variability in their body rotations at various aperture widths [28,29]. Patients with Parkinson’s disease (PD) showed sharply decreased walking speeds in front of an aperture, which could be caused by episodes of freezing [18]. When young adults used a manual wheelchair for the first time, contact with the frame of an aperture occurred more frequently with dramatically different spatial-temporal patterns of fixation [30,31]. To our knowledge, there has been no study testing the ability of stroke individuals to safely walk through an aperture.

Measuring the behavior of walking through an aperture potentially provides some new insights into the increased risk of instability during adaptive locomotion in stroke individuals. This is particularly because stroke individuals could show difficulty performing the body rotations due to their motor paralysis on one side of their body and, as a result, they could have difficulty avoiding accidental contact with the frame of apertures. Walking through a narrow aperture with body rotation results in unique postural configurations, i.e., the body is rotated in the yaw dimension while the walking direction is maintained toward the center of the aperture. The uniqueness of the body rotation behavior becomes clear when it is compared with the turning behavior, which also involves individuals rotating their bodies, while its purpose is to change the direction of walking. Rotating the body to walk through an aperture usually involves a pivot-like turn, in which the body rotates about its vertical axis on the trailing limb at the moment it crosses the aperture. If stroke individuals penetrate an aperture from the paretic side (i.e., the trailing limb is non-paretic), then they would be able to perform a pivot-like turn. However, they would have difficulty maintaining their balance after the turn because they need to shift their body weight to the leading, or paretic, limb to progress forward. In contrast, if stroke individuals penetrate an aperture from the non-paretic side (i.e., the trailing limb is paretic), then an alternate strategy, rather than a pivot-like turn, would be selected for rotating their bodies. In both cases, taking multiple steps to rotate the body, which has been observed in the turning behavior performed of older adults [32,33], was expected to occur. This was because it was effective, at least for stroke individuals, to avoid shifting their body weight onto the paretic limb for a relatively long time. However, because there has been no study, it remains unknown as to which strategy would be more preferable for strong individuals and which strategy would lead to safe walking through apertures without making any contact with the frame of an aperture. The rationale for conducting the present study was to clarify these issues.

In the present study, two groups of stroke individuals were recruited: stroke fallers and stroke non-fallers. Stroke fallers were identified as those having a history of falling history in the past year. A systematic review of the literature showed that a history of falling in the past year most strongly predicts the likelihood of future falls among community-living older adults [34]. Several previous studies have shown that significant gait characteristics of stroke individuals were more evident in those at high risk of falling [11,35]. Moreover, Takatori et al.(2009) reported that stroke individuals with a history of falls showed a large gap between the visual estimation of a reachable distance and the actual distance reachable [36]. If such a large gap between perception and action exists in various types of behavior, then stroke fallers would show inaccurate judgment of the passability of an aperture, which could lead to accidental contact with the frame of an aperture. To examine whether accidental contact was related to the inaccurate judgment of the passability of the aperture, participants in the present study performed both the behavioral task of walking through apertures and the perceptual judgment task of aperture passability.

## Methods

### Participants

**Fig 1:**
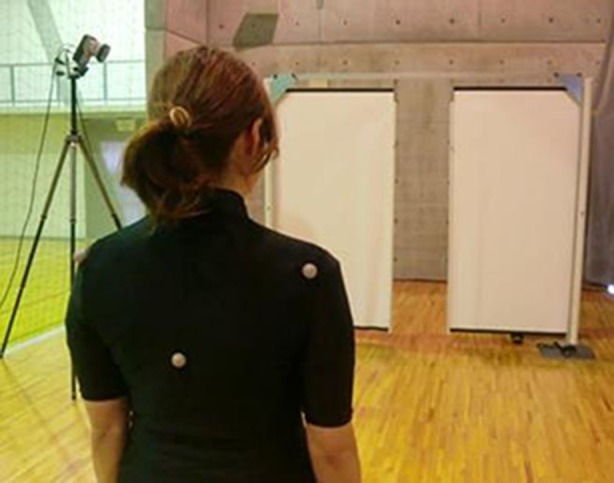
An experimental task. A participant walks toward a door-like aperture. The individual shown in Fig 1 has given written informed consent (as outlined in PLOS consent form) to publish an image of the participant.

Twenty-three individuals with stroke (eleven females) participated. The mean age was 60.7 years (SD = 10.1). Twenty-three age-, gender-, and height-matched healthy individuals also participated as control participants. This study was approved by the ethics committee of the Kameda Medical Center. The tenets of the Declaration of Helsinki were followed. All participants gave their written informed consent prior to participation. Notably, the individual shown in Fig 1 has given written informed consent (as outlined in PLOS consent form) to publish an image of the participant.

Participants in the stroke group were patients in a subacute hospital or had been discharged from a subacute hospital. The mean time from the onset of stroke to testing was 15.2 ± 21.1 months (ranging from 1 to 78 months). Participants had residual hemiparesis. The inclusion criteria ensured that participants had been walking for at least one month after a first-time stroke and that they were able to walk independently for more than 100 m with or without an assistive device. The exclusion criteria ensured that none of the participants had any indications of the following symptoms: (a) neurological, orthopedic, or other disorders that could affect walking, (b) history of visual deficits, (c) visual field deficits and visual spatial neglect, and (d) a score of less than 24 on the Mini Mental State Examination (MMSE) [37].

All stroke participants were asked whether they had fallen in the past 12 months. A fall was defined as an event that results in a person coming to rest unintentionally on the ground or other lower level [38]. Falls resulting from uncommon environmental factors (e.g., traffic accidents or while riding a bicycle) were excluded. Based on this definition, 10 participants were referred to as *stroke fallers* in this study. Six of these participants had fallen two or more times. The other 13 participants were referred to as *stroke non-fallers* in this study. Characteristics of participants are summarized in Tables 1 and 2 (see “Procedure and tasks” for detailed information about the clinical measurements described in Tables 1 and 2).

**Table 1:** Characteristics of participants.

	Stroke fallers	Stroke non-fallers	Control participants	p value
	(n = 10)	(n = 13)	(n = 23)	
**Participant details**				
** Gender (male/female)** ^**b)**^	6 / 4	6 / 7	11 / 12	n.s
** Age (y)** ^**a)**^	63.1 ± 9.0	58.8 ± 10.8	61.0 ± 9.7	n.s
** Height (cm)** ^**a)**^	160.0 ± 20.9	158.8 ± 10.9	160.9 ± 9.0	n.s
** Shoulder width (cm)** ^**a)**^	45.7 ± 4.0	46.7 ± 4.7	44.4 ± 2.3	n.s
** Minimum passable width**^**a)**^	1.13 ± 0.05	1.09 ± 0.04	1.08 ± 0.04	0.048*
**Clinical tests**				
** MMSE**^**a)**^	28.7 ± 1.9	28.8 ± 1.9	29.6 ± 0.9	n.s
** TUG (s)** ^**a)**^	16.8 ± 6.4	11.6 ± 5.5	6.23 ± 0.9	< 0.001*†
**Stroke participants’ details**				
** Time after stroke (months)** ^**c)**^	19.4 ± 21.5	12.0 ± 21.1		n.s
** mRS (score 2/score3)** ^**b)**^	3 / 7	10 / 3		n.s
** Lower extremity BRS (stages Ⅲ/Ⅳ/Ⅴ)** ^**b)**^	1 / 7 / 2	0 / 6 / 7		n.s
** Stroke type (hemorrhagic/ischemic)** ^**b)**^	5 / 6	6 / 7		n.s
** Hemiplegic side (right/left)** ^**b)**^	5 / 6	8 / 5		n.s
** Use of walking aid (no/yes)** ^**b)**^	6 / 4	7 / 6		n.s

a) Kruskal-Wallis test.

b) Pearson’s chi-square test.

c) Mann-Whitney U test. Note. MMSE = Mini Mental State Examination, BRS = Brunnstrom Recovery Stage, mRS = modified Ranking Scale. Minimum passable width is the ratio between the aperture width and the participant’s shoulder width.

* Significant difference between stroke fallers and control participants.

† Significant difference between stroke non-fallers and control participants.

**Table 2:** Participant information.

		Matched	Time			BRS	Mean			Mean
	Gender/	controls	since			Lower	TUG	Use of	modified	relative
	Age	Gender/	stroke	Paretic	Fall	extremity	time	walking	Ranking	perceptual
Participant	(years)	Age	(months)	side	history	score	(s)	aid	Scale	boundaries
1	M/40	M/43	67	Right	Faller	4	15.5	SPS	3	1.15
2	F/72	F/71	12	Left	Faller	4	14.5	AFO	3	0.81
3	F/66	F/67	50	Right	Faller	3	31.8	AFO, SPS	3	0.83
4	M/64	F/64	5	Left	Faller	4	18.3	AFO, SPS	3	0.92
5	F/66	F/68	14	Left	Faller	4	19.1	None	3	1.03
6	M/64	M/63	3	Left	Faller	5	20.3	SPS	3	0.92
7	F/66	F/61	12	Left	Faller	5	10.2	SPS	2	1.02
8	M/59	M/61	12	Right	Faller	4	7.7	None	2	1.1
9	M/62	M/62	16	Right	Faller	4	10.3	AFO	2	0.82
10	M/72	M/74	3	Right	Faller	4	16.1	SPS	3	1.06
11	F/48	F/48	78	Left	None	4	16.9	AFO, SPS	3	0.96
12	F/70	F/69	2	Right	None	5	10.2	None	2	0.96
13	M/56	M/53	1	Right	None	5	6.5	None	2	1.14
14	F/43	F/45	2	Left	None	5	7.2	None	2	1.09
15	F/62	F/61	4	Right	None	4	12.3	None	2	1.31
16	M/56	M/61	3	Right	None	4	11.1	AFO, SPS	3	0.94
17	F/75	F/75	9	Right	None	4	25.3	SPS	2	1.27
18	F/70	F/70	10	Left	None	4	12.5	SPS	2	0.97
19	M/69	M/69	2	Right	None	5	7.4	None	2	1.13
20	M/53	M/50	21	Right	None	4	13.7	AFO, SPS	2	0.73
21	M/64	M/67	21	Right	None	5	10.6	SPS	2	1.02
22	M/41	M/43	1	Left	None	5	5.2	None	2	0.77
23	F/58	F/60	2	Left	None	5	10.4	SPS	3	1.04

SPS (Single-point stick). AFO (Ankle Foot Orthosis).

### Apparatus

The experiment was performed along a straight 6.0-m path. A door opening was located 4.0 m in front of the location where participants started walking. A door-like aperture was created as a space between two projector screens (see Fig 1). Each screen was attached with an aluminum frame (2.2 m wide × 2.0 m high) so that the screens were located perpendicularly to the floor. The width of an aperture was easily adjustable by changing the location of the edge of each screen. After finishing the adjustment, the upper edge of each screen was fixed to the horizontal aluminum frame so that the width of an aperture remained immobile. Upper-body kinematics were measured with a three-dimensional motion analysis system (OQUS 300, Qualisys, Sweden) at a sampling frequency of 60 Hz. Six cameras tracked three passive retroreflective markers attached to participants: two markers each for the left and right shoulders (the lateral border of the spine of the scapula) and one marker for the spinous process of the 7^th^ thoracic vertebrae (T7). Two additional markers were placed on the inner edge of the doorframes to measure the position of the door opening.

### Procedures and tasks

The experiment consisted of three parts, which included (a) taking clinical measurements and some measurements of participants’ characteristics, (b) performing the task of walking through an aperture, and (c) performing a perceptual judgment task regarding aperture passability. Clinical measurements and measurements of participants’ characteristics were conducted first; however, the measurement of the minimum passable width was conducted after performing the two tasks to avoid the possibility that the experience of measuring could affect their performance in these tasks. The order of performing the two tasks was counterbalanced.

#### Walking through the aperture

A main experimental task was walking through apertures of various widths. Participants were asked to approach and walk through an aperture without making any contact with a screen. There were five different aperture widths: 0.9, 1.0, 1.1, 1.2, and 1.3 times the width of participants’ shoulders. To ensure participants’ safety while they were attempting to pass through an aperture, two therapists stood beside the door apparatus while participants performed the task.

Notably, aperture widths were relative to shoulder width for all participants, regardless of whether participants used the stick. Holding the stick itself did not alter the minimum passable width because participants were able to adaptively change the position of the hand holding the stick. We confirmed that our aperture width settings did not create any disadvantage for stroke individuals using a stick by conducting a preliminary analysis showing that there was no significant difference in the contact rate between stroke individuals who used a single-point stick and those who did not (*F* (1,21) = 2.45, *ns*).

For each trial, participants stood at the starting position (i.e., 4 m in front of the aperture) while visual information about the aperture was occluded by a large plate placed 20 cm in front of them. After the width of the aperture was adjusted, the visual occlusion was removed. Participants then started walking. They were allowed to rotate their bodies when necessary to avoid contact.

Prior to performing the main trial, participants performed three pretrial practices to familiarize themselves with the task. Aperture widths narrower than 1.1 times their shoulder widths were presented in at least two of these three trials. The order of the aperture width presented was randomized. After the pretrial practice, participants performed a total of 15 main trials (three trials for each of five aperture widths). The order of the aperture widths presented during the 15 trials was randomized. Participants were able to rest between trials when they felt it to be necessary.

#### Perceptual judgment of aperture passability

Another experimental task was to judge the passability of an aperture. Participants stood 3 m in front of an aperture. The floor was covered with a white cloth to prevent participants from obtaining any information from the floor around the door apparatus to aid in estimating the width of the aperture. They observed apertures of various widths and reported whether they believed they would be able to pass though the aperture without body rotation. A series of apertures was presented to participants using the staircase method. In this method, a series of opening widths was presented in either an ascending or descending order with consecutive 2-cm intervals. The presentation of the ascending (descending) series was started with an aperture selected randomly from those that were 15–20 cm wider (narrower) than the width of participants’ shoulders. The presentation of the series was terminated when the participants alternated their response (i.e., from passable to impassable, or from impassable to passable) a total of six times. Each participant performed one ascending series and one descending series. They were required to close their eyes during the intervals between the trials, during which the size of the aperture was changed. After finishing this task, participants’ actual minimum passable widths were measured. The actual minimum passable width was defined as the minimum width that a participant could pass through the aperture twice consecutively without body rotation. Minimum passable width is the ratio between the aperture width and the participant’s shoulder width.

#### Clinical measurements

Characteristics of participants (gender, age, and height) and clinical documentation of stroke participants (stroke type, side of lesion, and date of stroke) were obtained through medical records. The mean body width at the shoulders was defined as the distance between the heads of the right and left humeri. Two clinical measurements were conducted. Participants’ cognitive function was assessed with the Mini Mental State Examination (MMSE, Holsinger et al. 2007). Functional mobility was assessed with the Timed Up and Go (TUG) test (Ng and Hui-Chan 2005). In the TUG, participants were instructed to stand up from a standard chair with a seat height of 43 cm, walk a distance of 3 m at a maximum speed, turn, walk back to the chair, and sit down. To describe the impairment and activity limitation in stroke participants, the modified Ranking Scale (mRS) (van Swieten et al. 1988), which assesses the limitation of activity, and the lower extremity Brunnstrom Recovery Stages (BRS) (Brunnstrom 1966), which assesses the degree of recovery of lower extremity mobility, were determined. The mRS scores ranged from 0 (no symptoms at all) to 5 (severe disability: bedridden, incontinent, and requiring constant nursing care and attention). The BRS ranged from stage 1 (flaccidity: capable of no voluntary movement on the most-affected side) to stage 6 (spasticity disappears except for when fatigued; (movement of individual joints is almost normal). These measurements were performed in random order.

### Dependent measures and statistical analyses

#### Characteristics of participants, clinical measurements, and stroke participant details

The characteristics of participants (gender, age, height, shoulder width, and minimum passable width) and the results of clinical tests (MMSE and TUG) were compared statistically among the three groups (stroke fallers, stroke non-fallers, and control participants). A non-parametric test, the Kruskal-Wallis test, was used as the statistical test for all but gender. A significant main effect in the Kruskal-Wallis test was analyzed further using the Mann-Whitney U test with Bonferroni corrections. A Pearson’s chi-square test was used for gender. Statistical comparisons between stroke fallers and stroke non-fallers were also made in terms of the time since the stroke, mRS, lower extremity BRS, stroke type, hemiplegic type, and the use of a walking aid. A Mann-Whitney U test was used to measure the time since the stroke, whereas a Pearson’s chi-square test was used for the other measurements. The software package SPSS (version 21.0) was used.

#### Walking through an aperture

Dependent measures were categorized as one of two types: contact with the frame of an aperture and kinematic characteristics at the moment of aperture crossing.

To explain the characteristics of contact with the frame of an aperture, we calculated the contact rate, i.e., the percentage of contacts with the door’s edges in 15 trials; the contact frequency based on the number of contacts, i.e., no contact, single contact, or multi contacts; and the contact frequency based on the body side where the contact occurred. Contact with the frame of an aperture was detected by three experimenters. To correctly detect contact, participants were asked to inform experimenters whenever they touched the door. Contact frequency according to body side where the contact occurred, was measured separately in three situations of body rotations: no rotation, penetration from the paretic side, and penetration from the non-paretic side. The distribution of the percentage data and the number of steps were not normal. Therefore, we transformed the data used in the current study (i.e., the percentage of contact and the number of steps necessary to cross an aperture) by means of angular transformation (also known as arcsine transformation) prior to performing a two-way ANOVA. A Pearson’s chi-square test was used to analyze the contact frequency and the side of the body where contact occurred.

Kinematic characteristics at the moment of aperture crossing were described in terms of five measurements: the absolute body rotation angle, the number of steps necessary to cross an aperture (usually representing the steps necessary for body rotation), the body side to penetrate an aperture, the movement speed, and the absolute deviation of the upper-body midpoint from the center of the doorway. We included all measurements from the three trials in each experimental condition, even if contact occurred. Because the screen was soft, a contact did not drastically alter the kinematic characteristics. Moreover, we used the kinematic measurements was obtained at the moment of aperture crossing. The moment of aperture crossing was defined as the moment at which a passive retroreflective marker attached to left or right marker crossed the aperture. With this definition, a contact occurred in many cases after the kinematic measurement had already been obtained.

The body rotation angle in the yaw dimension was defined as the angle created between the aperture, represented by the two reflective markers on the edges of the screen, and the body, represented by two markers on the left and right shoulders (values larger than zero indicate counterclockwise rotation). Absolute values of the rotation angles were used as dependent measures.

The steps necessary to cross an aperture at the moment of crossing the aperture were counted with two types of video images that captured a participant’s body-rotation behavior in the frontal or sagittal dimension. Three volunteers who were not involved in collecting the data performed this analysis. Three volunteers watched the videos and counted the number of steps to cross an aperture. If no consistent results were obtained among them, then they watched the videos again together and determined the number. For trials in which body rotation to avoid contact occurred, this measurement represented the number of steps necessary to accomplish body rotation. For the trials in which no body rotation occurred, this measurement represented the number of steps taken to simply cross the aperture and was always regarded as a single step in this study. Based on previous studies [17,26], the beginning of body rotation was defined as the moment that the body rotation angle deviated from the mean body rotation angle for an initial 1 sec of measurement by more than three standard deviations (i.e., the mean ± 3 SD). The end of body rotation was defined as the time when the upper body (represented by the midpoint of the two markers on the shoulders) crossed the midpoint of the aperture.

The body side that penetrated the aperture was checked only for stroke fallers and stroke non-fallers because the purpose of this measurement was to investigate whether stroke participants penetrated from the paretic side or non-paretic side when body rotation occurred. Considering that rotation mostly occurred for narrow apertures, the penetrating body side was measured for relatively narrow apertures (relative aperture widths were 0.9, 1.0, and 1.1 times participants’ shoulder widths). As exploratory examinations looking for factors that could affect which body side penetrates an aperture, we compared the frequency of penetration between stroke fallers and non-fallers, and between participants whose lower extremity BRS stage was 5 and those whose BRS stage was 3 or 4.

For all measurements, except which body side penetrated an aperture, statistical analysis was conducted using a group (stroke fallers, stroke non-fallers, or age-matched controls) × an aperture width (0.9, 1.0, 1.1, 1.2, and 1.3 times the shoulder width) with repeated measures analysis of variance (ANOVA) of aperture width. Partial eta-squared values (*η*_*p*_^*2*^) were calculated as an unbiased estimate of the effect size in an ANOVA. Significant main and interaction effects in the ANOVA were analyzed further using Bonferroni-corrected pairwise comparisons. A Pearson’s chi-square test was used to statistically analyze the body side that penetrated the aperture for each comparison.

#### Perceptual judgment of aperture passability

The dependent variable was the perceived minimum passable width relative to the minimum passable width (referred to as the *relative perceptual boundary*). The perceived minimum passable width was calculated as the average of twelve aperture-width values with which participants alternated their responses in each stage of an ascending and descending series (i.e., six values were obtained from each series). The relative perceptual boundaries were analyzed in a one-way (group) ANOVA. A one-sample t-test was also carried out to examine whether the results of each condition would be significantly different from a value of 1.0. A value of 1.0 meant that their judgment was accurate, and values smaller than 1.0 meant that participants overestimated their passability (i.e., they underestimated the space necessary for passage).

It should be noted that the order of performing the two tasks was counterbalanced. With this procedure, it is possible that participants who performed the walking task first had an advantage for performing the perceptual judgment task. However, we confirmed that there was no significant difference in the perceptual judgments between those who performed the perceptual judgment task first and those who performed the walking task first (*t* (44) = 1.31, *ns*).

## Results

### Characteristics of participants, clinical measurements, and stroke participants’ details

With regard to the characteristics of participants and clinical measurements, a significant main effect of the group was found in the minimum passable width (*H* = 6.09, *p* = 0.048, *η*_*p*_^*2*^ = 0.14) and the TUG (*H* = 27.16, *p* < 0.001, *η*_*p*_^*2*^ = 0.60). Multiple comparisons with Bonferroni corrections showed that the minimum passable widths were significantly wider for stroke fallers than for controls. The time required for the TUG was significantly slower for stroke fallers and non-fallers than for controls. There were no significant differences among the three groups regarding other characteristics of participants and clinical measurements. Measurements of the stroke participants’ details showed that each mRS score was either 2 (slight disability: unable to carry out all previous activities but able to look after own affairs without assistance) or 3 (moderate disability: requiring some help but able to walk without assistance). Because our inclusion criteria in the present study ensured that participants were able to walk independently for more than 100 m, no participants were evaluated as having scores of 4 and 5. No significant main effect of the group was found in mRS (*U* = 34.50, *p* = 0.057, *r* = 0.37). The lower extremity BRS was either stage 3 (spasticity increases: gaining voluntary control of movement in synergy patterns), stage 4 (spasticity decreases: the beginning of voluntary movement without synergy patterns), or stage 5 (spasticity continues to decline: capable of more complex natural movements) for all participants. No significant main effect of the group was found in the BRS (*U* = 40.00, *p* = 0.13, *r* = 0.46).

### Walking through the aperture

**Fig 2:**
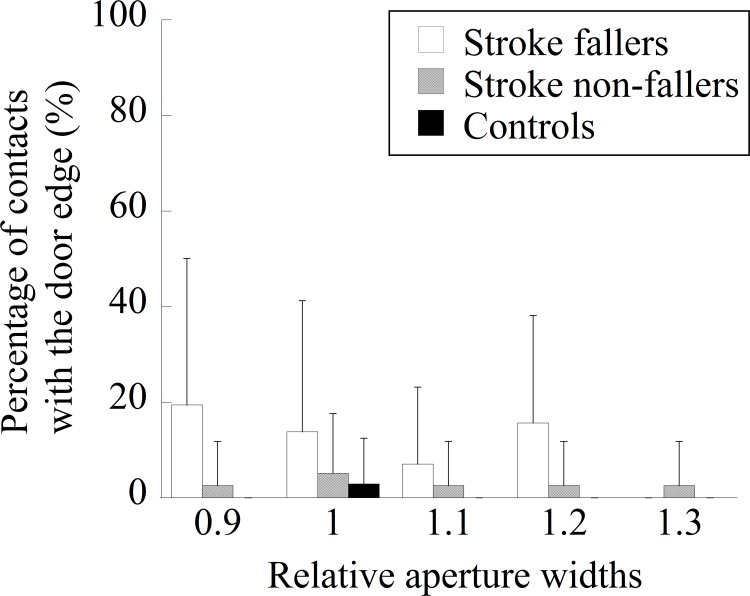
The mean percentage of contacts with the frame of an aperture in each group.

The mean contact rate in each group is shown in Fig 2. The main effect of the group was significant (*F* (2, 43) = 21.18, *p* < 0.001, *η*_*p*_^*2*^ = 0.50). Post-hoc multiple comparisons showed that the mean contact rate was significantly higher in stroke fallers than in stroke non-fallers and control participants. The main effect of the aperture width was significant (*F* (4, 172) = 4.81, *p* = 0.0011, *η*_*p*_^*2*^ = 0.10). The percentage was significantly higher when the relative aperture widths were 0.9 and 1.0 than when it was 1.3. The interaction between the group and the aperture size was also significant (*F* (8, 172) = 3.18, *p* = 0.0022, *η*_*p*_^*2*^ = 0.13). When the aperture width was relatively narrow (i.e., 0.9 and 1.0 times the participant’s shoulder width), stroke fallers experienced contact significantly more frequently than did stroke non-fallers and controls. When the relative aperture width was 1.1, stroke fallers experienced contact significantly more frequently than did controls.

Table 3 shows the contact frequency, classified as no contact, single contact, or multi contacts, in stroke fallers and non-fallers. A chi-squire analysis showed a significant difference (*χ2* (2) = 10.57, *p* = 0.0051). Multiple contacts with the door edges were made by 6 of 22 stroke participants. In addition, six people who had multiple contacts all were fallers.

**Table 3:** Contact frequency classified as no contact, single contact, or multi contacts.

	No contact	Single contact	Multi contacts
Stroke fallers	2	2	6
Stroke non-fallers	7	6	0

**Fig 3:**
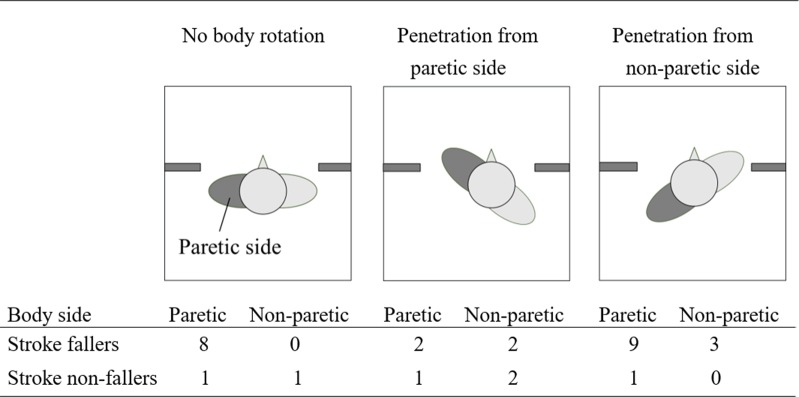
Contact frequency classified according to the body side where contact occurred.

Fig 3 shows the contact frequency classified according to the frequency of the body side where contact occurred in stroke fallers and non-fallers. Stroke participants performed 117 trials with no body rotation, 115 trials with penetration from the paretic side, and 113 trials with penetration from the non-paretic side. The expected values of the chi-square test were all equal, because all three rotation patterns occurred roughly with the same frequency. A chi-square analysis showed a significant difference in stroke fallers (*χ2* (5) = 15.50, *p* = 0.0056) but not a significant difference in stroke non-fallers (*χ2* (5) = 2.00, *p* = 0.85). Multiple comparisons with Bonferroni corrections showed that, for stroke fallers, contact occurred more frequently in the affected side of the body when participants showed no body rotation and showed penetration from the non-paretic side. For stroke non-fallers, there was no significant difference regarding the body side where contact occurred.

**Fig 4:**
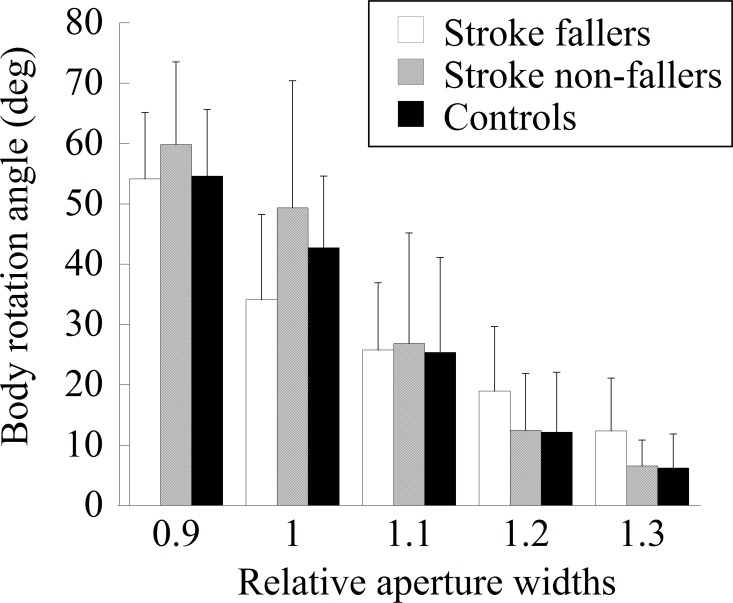
The mean absolute angle of body rotation for each aperture width in each group.

The mean absolute angles of body rotation for all aperture widths in each group are shown in Fig 4. The main effect of the group was not significant (*F* (2, 43) = 0.31, *ns*). The main effect of the aperture width was significant (*F* (4, 172) = 204.21, *p* < 0.001, *η*_*p*_^*2*^ = 0.83). Multiple comparisons showed that the absolute angle of body rotation of each pair of five aperture widths was significantly different. A significant interaction between the two factors (*F* (8, 172) = 3.75, *p* < 0.001, *η*_*p*_^*2*^ = 0.15) indicated that the absolute body rotation angle was significantly smaller in stroke fallers than in stroke non-fallers when the aperture width was 1.0.

**Fig 5:**
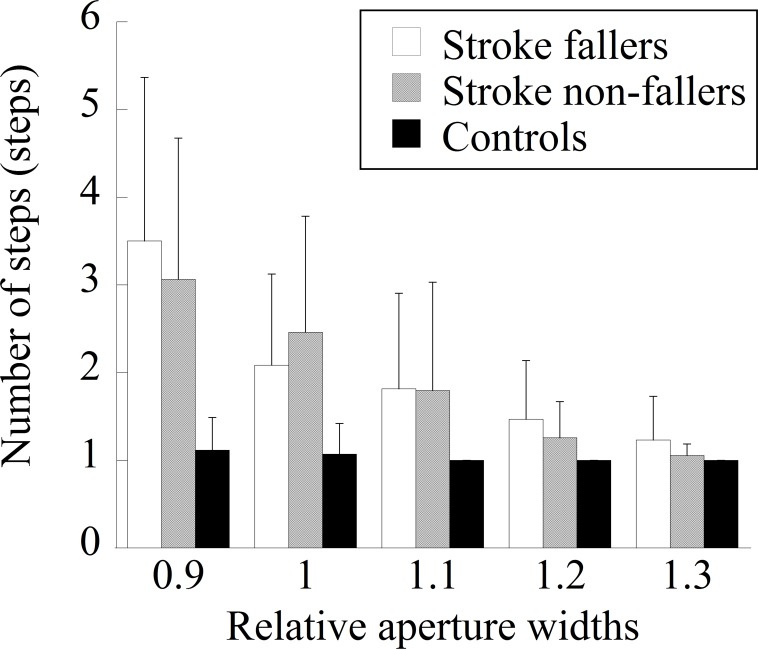
The number of steps necessary to cross the aperture in each group.

The steps necessary to cross an aperture are shown in Fig 5. The main effect of the group was significant (*F* (2, 43) = 11.64, *p* < 0.001, *η*_*p*_^*2*^ = 0.35). The number of steps necessary to cross an aperture was significantly larger for stroke fallers and non-fallers than for control participants. The main effect of the aperture width was significant (*F* (4, 172) = 52.04, *p* < 0.001, *η*_*p*_^*2*^ = 0.55). With the exception of the comparison between widths of 1.2 and 1.3, the numbers of steps were significantly different between each pair of five aperture widths (i.e., larger steps for narrower aperture widths). There was significant interaction between the two factors (*F* (8, 172) = 11.63, *p* < 0.001, *η*_*p*_^*2*^ = 0.56). Multiple comparisons showed that, when the aperture size was relatively narrow (i.e., 0.9, 1.0, and 1.1 times the participant’s shoulder width), more steps were necessary for stroke fallers and stroke non-fallers. For stroke fallers, the number of steps was significantly larger than other widths when the relative aperture width was 0.9. The number of steps was also larger when the relative aperture width was 1.0 than when it was 1.2 or 1.3, and when the relative aperture width was 1.1 than when it was 1.3. For stroke non-fallers, the number of steps between each pair of five aperture widths was significantly different.

**Fig 6:**
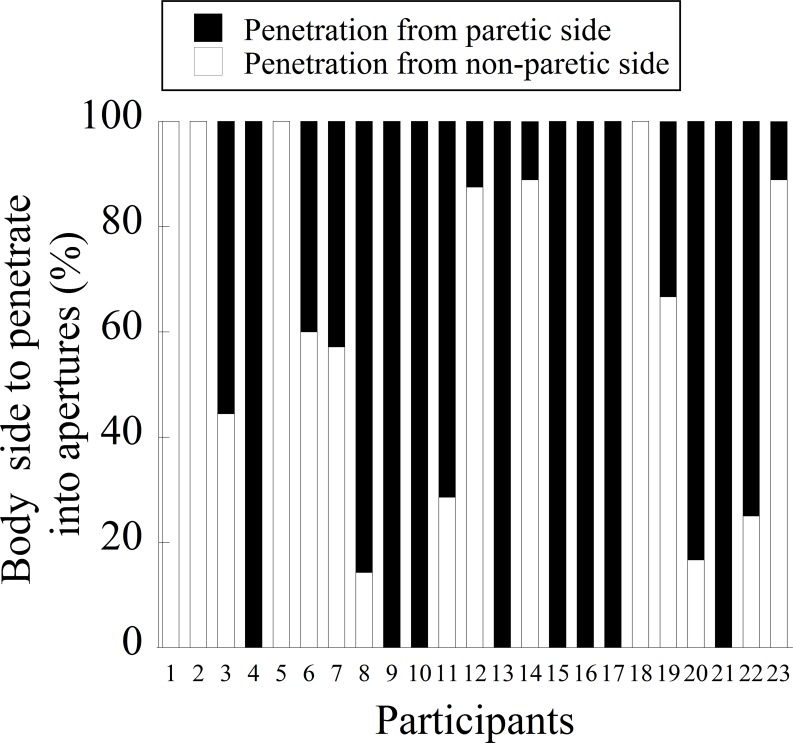
Frequency with which the body side penetrated an aperture in stroke fallers and non-fallers.

The frequency with which each body side penetrated a narrow aperture (i.e., 0.9, 1.0, and 1.1) for each stroke participant is shown in Fig 6. Except for four participants (Ss. 3, 6, 7, and 19), participants had preferences regarding with which body side to penetrate a narrow aperture. Twelve of 23 stroke participants (52%) penetrated an aperture with one side of the body throughout the 15 trials. As a whole, penetration of an aperture from the non-paretic side was 45.8%, and penetration from the paretic side was 54.2%.

Table 4 shows the frequency with which the body side penetrated the aperture in stroke fallers and non-fallers. A chi-squire analysis showed no significant differences (*χ2* (1) = 0.31, *p* = 0.45). Table 5 shows the frequency of each body side penetrating the aperture in participants with a lower extremity BRS of 5 and those with a BRS of 3 or 4. Although participants whose BRS scores were 3 and 4 seemed to show more frequent penetration of a narrow aperture from the paretic side, a chi-square analysis failed to show a significant difference (*χ2* (1) = 3.24, *p* = 0.086).

**Table 4:** The frequency of each body side penetrated the aperture in stroke fallers and non-fallers.

	Fallers	Non-fallers
Penetration from the paretic side	5	8
Penetration from the non-paretic side	5	5

**Table 5:** Frequency of body side penetrating an aperture in participants with a lower extremity BRS of 5 and those with a BRS of 3 or 4.

BRS lower extremity score	3 or 4	5
Penetration from paretic side	10	3
Penetration from non-paretic side	4	6

The mean of the maximum movement speed at the time of the aperture crossing is shown in Table 6. The main effect of the group was significant (*F* (2, 43) = 35.9, *p* < 0.001, *η*_*p*_^*2*^ = 0.63). Multiple comparisons showed that the maximum movement speed at the time of crossing was significantly slower in stroke fallers and non-fallers than in control participants. The main effect of the aperture width was also significant (*F* (4, 172) = 30.0, *p* < 0.001, *η*_*p*_^*2*^ = 0.41). The maximum movement speed at the time of aperture crossing was significantly different between each pair of five aperture widths with the exception of those between 1.2 and 1.3. Interaction between the two factors was not significant (*F* (8, 172) = 1.38, *ns*).

**Table 6:** The mean of movement speed at the time of aperture crossing (cm/s) (SD in parenthesis).

Aperture width					
(relative to shoulder width)	0.9	1	1.1	1.2	1.3
Stroke fallers	44.9 (19.6)	48.8 (19.0)	54.7 (18.5)	57.2 (22.8)	55.9 (24.1)
Stroke non-fallers	57.0 (29.6)	64.8 (29.4)	70.5 (28.5)	77.7 (32.1)	79.4 (30.3)
Controls	118.7 (22.3)	120.2 (25.5)	126.8 (20.8)	130.3 (22.7)	131.5 (20.9)

The absolute deviation of the upper-body midpoint from the center of the doorway is shown in Table 7. The main effect of the group was not significant (*F* (2, 43) = 1.73, *ns*). The main effect of the aperture width was significant (*F* (4, 172) = 17.4, *p* < 0.001, *η*_*p*_^*2*^ = 0.29). The absolute deviation of the upper-body midpoint from the center of the doorway became significantly larger as the aperture width narrowed. There was a significant interaction between the two factors (*F* (8, 172) = 2.09, *p* = 0.039, *η*_*p*_^*2*^ = 0.09). When the relative aperture width was 1.3, the absolute deviation of the upper-body midpoint from the center of the doorway was significantly larger in stroke fallers than in stroke non-fallers and controls.

**Table 7:** The mean of the absolute deviation from the center of the aperture at the time of aperture crossing under each experimental condition (mm) (SD in parenthesis).

Aperture width					
(relative to shoulder width)	0.9	1	1.1	1.2	1.3
Stroke fallers	54.1 (27.2)	45.9 (31.7)	36.8 (23.7)	38.8 (23.5)	44.8 (26.3)
Stroke non-fallers	62.0 (27.9)	51.9 (31.3)	34.7 (23.7)	23.6 (15.9)	23.6 (16.1)
Controls	48.3 (23.7)	45.9 (22.9)	31.7 (26.8)	21.9 (17.2)	16.3 (9.4)

### Perceptual judgment of aperture passability

**Fig 7:**
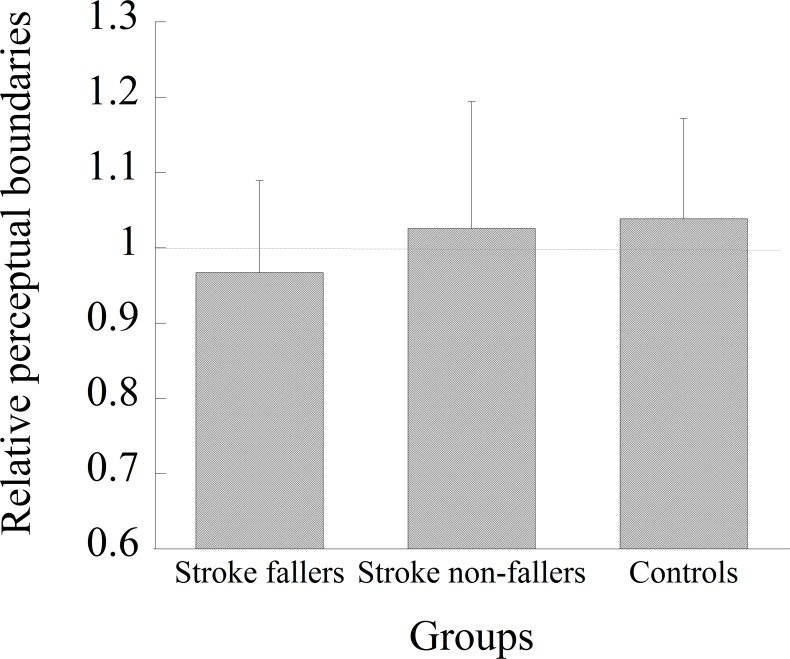
The mean relative perceptual boundaries obtained from the perceptual judgment task in each group.

Fig 7 shows the relative perceptual boundaries obtained for each group. An ANOVA showed no significant main effect of group (*F* (2, 43) = 0.88, *p* = 0.42). The one-sample t-tests showed that the relative perceptual boundaries obtained from each group were not significantly different from the value 1.0.

## Discussion

Contact with the frame of an aperture occurred more frequently in stroke fallers than in stroke non-fallers and control participants. Six of 10 stroke fallers made multiple contacts. In contrast, no stroke non-fallers made multiple contacts. These results suggest that, whereas stroke non-fallers who experienced accidental contact with the frame of an aperture successfully modified their behavior to avoid contact, many stroke fallers who experienced accidental contact could not. For stroke fallers, contact tended to occur more frequently on their affected side, whereas for stroke non-fallers, there was no tendency regarding the side of the body on which the contact occurred.

Previous studies have investigated whether maladaptive locomotion in obstacle avoidance was affected by perceptual-motor or locomotor factors in participants who had suffered a stroke and who had visuospatial neglect [39] and PD [18]. The findings in the present study suggest that accidental contact in stroke fallers was likely to occur mainly due to locomotor factors rather than perceptual factors. A comparison of participants’ characteristics showed that the only significant difference was found on the TUG. Stroke fallers performed the TUG more slowly (mean time was 16.8 ± 6.4 s), which showed their restricted mobility. The other basic characteristics relevant to stroke and activity limitation, such as the time since the stroke, stroke type, hemiplegic side, or mRS, were not significantly different between the two groups of stroke participants. Because our inclusion criteria in the present study ensured that participants had been walking for at least one month after a first-time stroke and that they could walk independently for more than 100 m, individual differences among the participants may have been relatively small. There was no significant group difference in the perceptual judgment of aperture passability (Fig 7). This suggests that the perceived aperture passability of stroke fallers was comparable to that of stroke non-fallers and control participants. In fact, this was consistent with a previous study that showed no relationship between the behavior of walking through an aperture and the perceptual judgment of aperture passability in participants with PD [18]. Considering these findings, the failure of stroke fallers to avoid contact is likely related to locomotor factors, i.e., the difficulty in modifying their behavior.

More specifically, the main reason for stroke fallers’ failure to avoid contact was likely insufficient body rotation. The measurement of the minimum passable width showed that stroke fallers required a wider space than did stroke non-fallers and control participants (Table 1). Given such a large minimum passable width, a larger magnitude of body rotation would be necessary for stroke fallers to avoid contact. However, the angle of body rotation at the moment of aperture crossing was not significantly different from that of other participant groups (Fig 4); rather, the body rotation angle was significantly smaller for the 1.0 aperture than those of other participant groups. Therefore, insufficient body rotation is likely to be a cause of frequent accidental contact.

Accidental contact in previous studies was considered to occur when fine-tuning of the walking path toward the center of an aperture was not successful [15,16]. In fact, the analysis of the deviation of the body’s midpoint from the center of an aperture showed that the deviation became larger for the 1.3 apertures for stroke fallers than for the other participant groups. However, because the stroke fallers showed no contact for the 1.3 aperture, the failure to fine-tune the walking path toward the center of an aperture could not be the reason for more frequent contact. Therefore, accidental contact in the present study did not result from the failure to fine-tune a walking path toward the center of an aperture.

Our particular interest in the present study was to understand the side of the body that penetrated an aperture and whether the selection of the body side for penetration was related to safe walking through apertures without making contact. Our results showed that there was no consistency among stroke participants on the body side to penetrate an aperture; eleven participants penetrated an aperture from the paretic side, while eight participants penetrated an aperture from the non-paretic side. There was no significant difference between stroke fallers and non-fallers. A visual inspection of Table 5 appeared to show that stroke participants with lower scores for their lower extremity BRS tended to show a preference for preference for penetration from the paretic side. However, this result did not reach a significant level.

Interestingly, the tendency in stroke fallers to make more contact on the paretic side disappeared when they penetrated an aperture from their paretic side. This result might be explained by two possibilities. First, vision may have been available to represent the paretic side of the body. While fixation is mainly directed toward a distant place during walking [31,40], visual information obtained from a lower visual field is important to perceive the spatial relationship between the environment and the body in the peri-personal space [41,42]. When stroke participants penetrated an aperture from their paretic side, the location of the paretic side of the body was visible through the lower visual field. Because the availability of proprioceptive inputs from the paretic side of the body is limited, the use of vision is helpful to represent the paretic side of the body and was, thus, helpful for perceiving the spatial relationship between the body and the aperture. Second, spatial attention may have been directed more toward the paretic side of the body. A previous study [43] demonstrated that, when walking through an aperture, the magnitude of the angle of body rotation is determined so that it creates a constant spatial margin between the frame of an aperture and the edge of the body side from which individuals penetrated the aperture. Providing that spatial attention is involved in information processing to produce the constant spatial margin, this could help improve the representation of the paretic side of the body. In fact, the attention was not sufficiently allocated during obstacle avoidance, contact occurred more frequently [44]. Future studies need to test the validity of these explanations. It would also be interesting to examine whether intervention to lead stroke individuals to penetrate an aperture from the paretic side would result in safe passage.

The results of the perceptual judgment task showed that the perceived aperture passability of stroke fallers was comparable to that of stroke non-fallers and control participants, at least when they observed an aperture from a remote place while standing still. However, it is still possible to assume that the perceptual judgment of stroke fallers may have been inaccurate if it was measured during walking. Hackney et al. have shown that age-related differences in action capabilities (i.e., older adults have more body rotation for wider apertures than do younger adults) were related to perceptual judgment under dynamic conditions (i.e., judgment of passability while walking toward an aperture) but not under static conditions (i.e., judgment of passability while standing still) [29]. According to this finding, future studies would need to examine whether stroke fallers would show inaccurate perceptual judgment under dynamic conditions. It is also possible to assume that, whereas stroke fallers were aware that contact could occur when the body was rotated as planned (i.e., their perceptual judgment was accurate), they did not change the strategy intentionally to avoid increased energetic/biomechanical costs with body rotation of a greater magnitude. On average, stroke participants (i.e., both fallers and non-fallers) needed three steps to cross the 0.9 aperture and two steps to cross the 1.0 aperture. Due to the necessity of taking multiple steps to rotate the body, energetic/biomechanical costs could increase while rotating the body [33]. In our experimental setting, participants were free from the risk of injury even if they made contact with the frame of an aperture because it was made of a soft material (a projector screen). Considering these situations, stroke fallers may have determined to avoid an energetic/biomechanical cost rather than avoiding contact.

## Conclusion

Stroke fallers, but not stroke non-fallers, showed frequent contact with the frame of an aperture. The failure to avoid contact was likely due to insufficient body rotation at the moment of aperture crossing, rather than the failure to fine-tune their walking path toward the center of an aperture. Because the perceived aperture passability was not significantly different among the three groups of participants, the insufficient body rotation did not simply result from inaccurate perceptual judgment performed from a remote place. Contact with the frame of an aperture occurred more frequently on the paretic side in stroke fallers. There was no consistency among stroke participants regarding the body side to penetrate an aperture. However, when they penetrated an aperture from the paretic side, contact on the paretic side did not occur frequently. Two possibilities explain this result, i.e., the availability of vision and/or the availability of spatial attention to represent the paretic side of the body.

## Supporting Information

S1 Table Detailed participant information and Results of three-dimension motion analysis. (XLSX)
